# Supplementary material for: Overexpression of a Malus baccata MYB Transcription Factor Gene MbMYB4 Increases Cold and Drought Tolerance in Arabidopsis thaliana
Source: Int J Mol Sci. 2022 Feb 4;23(3):1794. doi: 10.3390/ijms23031794 (PMC8836155; doi:10.3390/ijms23031794)
Supplement: Supplementary file 1 [file ijms-23-01794-s001.zip › ijms-1559158-supplementary.pdf]

Table S1 List of primers used in this study.

Primer Name	Primer Sequence (5'→3')	Purpose
MbMYB4-F	ATGGTTAGAACTCCTTGCCG	full-length cDNA of *MbMYB4*
MbMYB4-R	TCAAAATAGTGGATACAAAAACTCGG	full-length cDNA of *MbMYB4*
site-F	GCTCTAGAATGGATGCTTTCTCT	PCR for restriction site insertion
site-R	CGGGATCCAATGGAAAAACTC	PCR for restriction site insertion
MbMYB4-qF	TGCCGTGATGAAAATGGAAT	qPCR
MbMYB4-qR	TTTTTCGTGTAATTTGATGATAGTT	qPCR
Actin-F	ACACGGGGAGGTAGTGACAA	qPCR
Actin-R	CCTCCAATGGATCCTCGTTA	qPCR
HR-F	GGACAGGGTACCCGGGGATCCATGGTTAGAACTCCTTGCCG	PCR for homologous recombination
HR-R	CTGGCATGCCTGCAGGTCGACAAATAGTGGATACAAAAACTCGGAAT	PCR for homologous recombination
AtCBF1-F	TCGGGACTTTCCAAACCG	qPCR
AtCBF1-R	CCATCTCCTTCGCCGTCAT	qPCR
AtCBF3-F	TCCGGTAAGTGGGTTTGTGAG	qPCR
AtCBF3-R	AACTCGGCATCTCAAACATCG	qPCR
AtCOR15a-F	CAACAGAGGAATCACCAGCGA	qPCR
AtCOR15a-R	CTCTGCTGTCTTGTCGTGGTGT	qPCR
AtRD29a-F	CAACGAGGGGAAGATAAAAGTGT	qPCR
AtRD29a-R	AGCCAGATGATTTTGGAGCCT	qPCR
AtNCED3-F	ATGGCTTCTTCACGGCACGG	qPCR
AtNCED3-R	TTCCTTTGCCCTCGGACG	qPCR
AtSnRK2.4-F	GAGGAAATGGGGATGCAGAT	qPCR
AtSnRK2.4-R	TTCTCACTTCTCCACTTGCG	qPCR
AtCAT1-F	CGCCATGCCGAAAAATACCC	qPCR
AtCAT1-R	CTTGCCTGTCTGAATCCCAGGAC	qPCR
AtP5CS-F	GATACGGATATGGCAAAGCG	qPCR
AtP5CS-R	CCAAGTCCAAATCGGAAACC	qPCR
AtActin-F	CCCGCTATGTATGTCGC	qPCR
AtActin-R	AAGGTCAAGACGGAGGAT	qPCR

Table S2 List of physiological index measurement methods used in this study.

**Physiological Indicators**	**Calculation Formulas**	**Explanations**
Chlorophyll content	$Chl(g\cdot kg^{-1})=(12.7A_{663}-2.69A_{645})\ V/W+(22.9A_{645}-4.68A_{663})\ V/W$	A: absorbance value; V: total volume of reaction solution (mL); W: fresh weight of sample (kg).
Proline content	$Pro(\mu g/g)=C\cdot V/W\cdot v_1$	C: proline content calculated from the standard curve (μg); V: total volume of extract solution (mL); W: fresh weight of sample (g); v_1: volume of extract solution used in the measurement (mL).
Relative conductivity	$Rel= R_1/\ R_2\cdot 100\%$	R_1: leachate conductivity; R_2: leachate conductivity after cooling.
POD activity	$POD(U/gropt)=\Delta A\cdot V/0.01\cdot v_2\cdot \Delta t\cdot W$	ΔA: difference value in absorbance; V: total volume of reaction solution (mL); v_2: volume of enzyme solution added (mL); Δt: reaction response time; W: fresh weight of sample (g). Take the A_{470} drop by 0.01 in 1 min as 1 POD activity unit (U).
CAT activity	$CAT(U/gropt)=\Delta A\cdot V/0.01\cdot v_3\cdot \Delta t\cdot W$	ΔA: difference value in absorbance; V: total volume of reaction solution (mL); v_3: volume of enzyme solution added (mL); Δt: reaction response time; W: fresh weight of sample (g). Take the A_{600} drop by 0.01 in 1 min as 1 enzyme activity unit (U).
MDA activity	$MDA(nmol/g)=(\Delta A_{532}-\Delta A_{600})\cdot V\cdot v_4/1.55\cdot 10^{-1}\cdot W\cdot v_5$	ΔA_{532}: difference value between absorbance at 532 and 450; ΔA_{600}: difference value between absorbance at 600 and 450; V: total volume of reaction solution (mL); v_4: total volume of extract solution (mL); W: fresh weight of sample (g); v_5: volume of the extract solution added in the color reaction (mL).

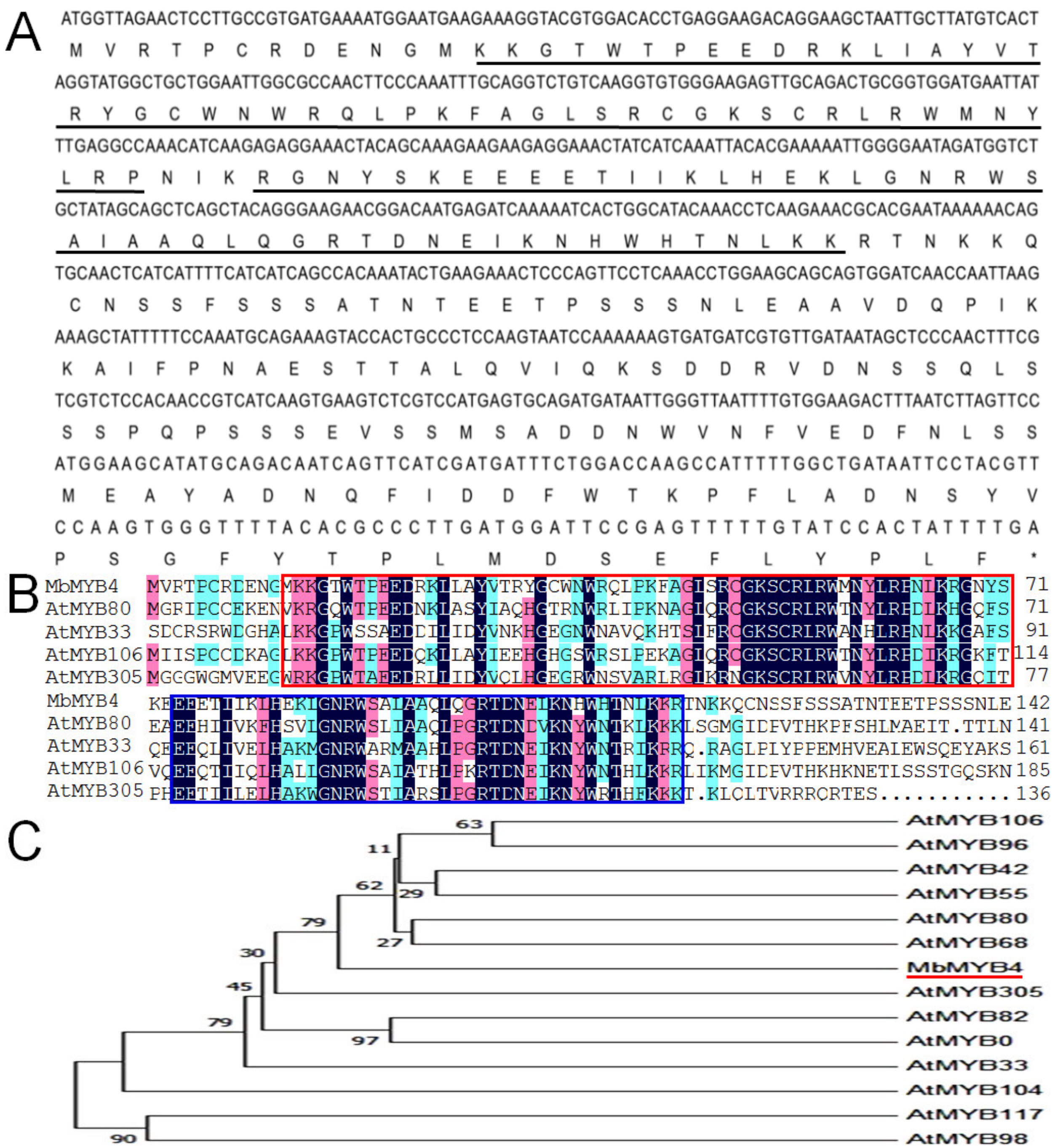

Figure S1. (**A**) Nucleotide and deduced amino acid sequences of *MbMYB4* gene. The conserved domain of R2 and R3 is underlined. (**B**) Comparison of homology between MbMYB4 and several *Arabidopsis thaliana* MYB proteins in order to point to the conservative domains. The sequence in the red and blue frame is the conserved amino acid sequence. (**C**) Phylogenetic tree analysis of MbMYB4 and *A. thaliana* MYB proteins. The accession numbers are as follows: AtMYB106 (*A. thaliana*, NP_186763.2), AtMYB96 (NP_851248.1), AtMYB42 (NP_567390.4), AtMYB55 (NP_001118913.1), AtMYB80 (NC_003076.8), AtMYB68 (NP_201380.1), AtMYB305 (AAB38777.1), AtMYB82 (NP_680426.1), AtMYB0 (NP_189430.1), AtMYB33 (NP_196228.1), AtMYB104 (NP_180263.5), AtMYB117 (NP_564261.1), AtMYB98 (NP_193612.1).

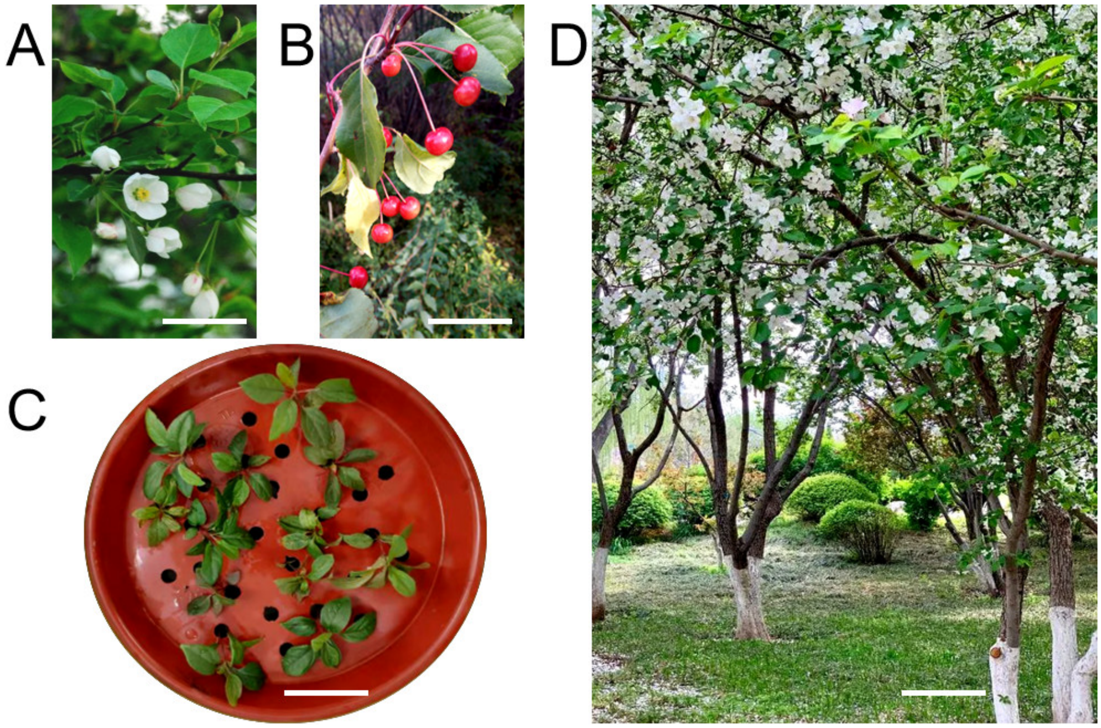

Figure S2. Flowers and twigs (**A**), fruits (**B**), hydroponic plantlets (**C**) of *Malus baccata*. Scale bars correspond to 5 cm. (**D**) Plants of *Malus baccata*. Scale bars correspond to 5 dm.
